# Exploring predictors of the five-time sit-to-stand test based on cross-sectional findings from the Swedish National Study on Aging and Care (SNAC)

**DOI:** 10.1186/s12877-025-05737-8

**Published:** 2025-02-04

**Authors:** Joakim Niklasson, Cecilia Fagerström, Sofia Backåberg, Patrick Bergman, Terese Lindberg

**Affiliations:** 1 Faculty of Health and Life Sciences, Linnaeus University, Kalmar, Sweden; 2 Department of Research, Region Kalmar County, Kalmar, Sweden; 3 Faculty of Health and Life Sciences, Linnaeus University, Växjö, Sweden; 4 Faculty of Kinesiology, University of Calgary, Calgary, Canada; 5 Faculty of Health and Life Sciences, Department of Medicine and Optometry, Linnaeus University, eHealth Institute, Kalmar, Sweden; 6 Department of Health, Blekinge Institute of Technology, Karlskrona, Sweden

**Keywords:** Aging, Balance, Physical activity, Physical function, Quality of life, Sedentary behavior, Sit-to-stand

## Abstract

**Background:**

As we age, staying physically active and reducing sedentary behavior becomes crucial. To understand how to achieve this, factors related to daily physical function such as five-time sit-to-stand (STS) time should be explored. This study aimed to investigate the associations between STS time, self-rated physical activity, physical function, health-related quality of life, physical and mental health in community-dwelling older adults aged ≥ 60 years.

**Method:**

Cross-sectional design with self-reported and objectively measured data from adults aged ≥ 60 years (*n* = 819), acquired from the Swedish National Study on Aging and Care. Data was analyzed through multiple linear regression.

**Results:**

The model (R^2^ = 0.268) showed that STS time was predicted by grip strength (β’ = -0.204, *p* < 0.05), age (β’ = 0.202, *p* < 0.05), health-related quality of life (β’ = -0.192, *p* < 0.05), having fallen within the preceding twelve months (β’ = -0.127, *p* < 0.05), physical activities of perceived light to moderate intensity (β’ = -0.121, *p* < 0.05), one-leg stand (β’ = -0.099, *p* < 0.05), and education level (β’ = -0.092, *p* < 0.05). For STS time, health-related quality of life (β = -0.354, confidence interval [CI] (-0.509)–(-0.199)), having fallen within the preceding twelve months (β = -0.222, CI (-0.365)–(-0.078)), and physical activities of perceived light to moderate intensity (β = -0.166, CI (-0.278)–(-0.053)) were the most prominent predictors.

**Conclusion:**

The model highlights the importance of grip strength and health-related quality of life in predicting STS time in older adults. Clinicians can use these insights to develop interventions that maintain physical function by regularly assessing and monitoring these factors. Future research should explore the relationship between fall history, faster STS time, and the impact of grip strength and health-related quality of life on sedentary behavior among older adults.

## Background

As we age, physical function declines, making it crucial for older adults to remain as physically active as possible. Furthermore, the importance preserving lower body strength and balance becomes vital measure for older adults health and to increase quality in life [1]. The physical activity recommendations for older adults includes: 150 min of moderate-intensity exercise (brisk walking) weekly, plus strength training and balance exercises [1]. Physical inactivity, defined by WHO [1] as not meeting these recommendations, becomes more common with age. Therefore, incorporating activity into daily routines is essential for optimal health. This is especially important as sedentary behavior naturally increases with age [1, 2].

In Sweden older adults aged 65–84 shows the highest age-related prevalence of sedentary behavior in leisure activities, which increases the risk of physical inactivity [1, 3]. Inactivity raises health problems and mortality risk. However, reducing time spent sitting time can lessen these risks [1]. This emphasizes the need for research that explore underlying structures of sedentary behavior, such as physical function.

The ability to rise independently from a seated position has long been a critical measure of lower body strength and overall physical function in older adults [4]. Inability to rise not only increases the risk of prolonged sitting and inactivity [5], but also reflects a decline in key performance factors like lower limb muscle control and balance [6]. Furthermore, a history of falls, often linked to the inability to stand, is a strong predictor of reduced independence in daily life [7]. Grip strength serves as another important indicator of physical function, acting as a long-established biomarker for overall health in older adults [8].

The debate regarding prolonged sitting among older adults is ongoing, and greater understanding of underlying factors that affect physical function such as the ability to stand up from sitting, is needed [9]. Investigating underlying factors of sit-to-stand time (STS) in older adults could reveal new ways to address sedentary behavior. This, in turn, could provide insights on how to increase physical activity in their daily lives. To our knowledge, there is a lack of research focusing on older adults’ STS time as a proxy measure of daily physical activity or sedentary behavior. As the five-time STS test provides a reliable measure for lower body physical function, its connection to the risk of prolonging sitting time becomes interesting. Therefore, this study sought to find predictors associated with STS time in community-dwelling older adults.

## Method

### Aim

To investigate the associations between STS time, self-rated physical activity, physical function, health-related quality of life, physical and mental health in community-dwelling older adults aged ≥ 60 years.

### Study design

Cross-sectional, descriptive research design.

### Participants and recruitment procedure

The study population was drawn from participants in the Swedish National Study on Aging and Care (SNAC) conducted at four research centers in Sweden: Malmö, Karlskrona, Stockholm, and Nordanstig [10]. SNAC is a longitudinal study in Sweden investigating aging and care. It follows a representative group of older adults to understand the aging process and how they receive care and services. The participants of SNAC are a representative sample of the Swedish population aged 60, 66, 72, 78, 81, 84, 87, 90, 93, or 96 years at inclusion. The purpose of SNAC is to improve the Swedish care system by providing valuable data for research and policy decisions [10].

The sample included data from three baseline surveys: Cohort 1 (C1) collected in 2003, Cohort 2 (C2) collected in 2009, and Cohort 3 (C3) collected in 2015. All individuals aged 60 years and above were included from each cohort, with unique identification codes used. At the start of C1, participants were aged 60, 66, 72, 78, or 81 years and older. For C2 and C3, new participants aged 60 or 81 years were enrolled. Data from C1-C3 were combined into a single cross-sectional dataset. All participants underwent clinical examinations and physical tests measuring physical function, such as grip strength and STS time, and self-reported health [10].

**Fig. 1:**
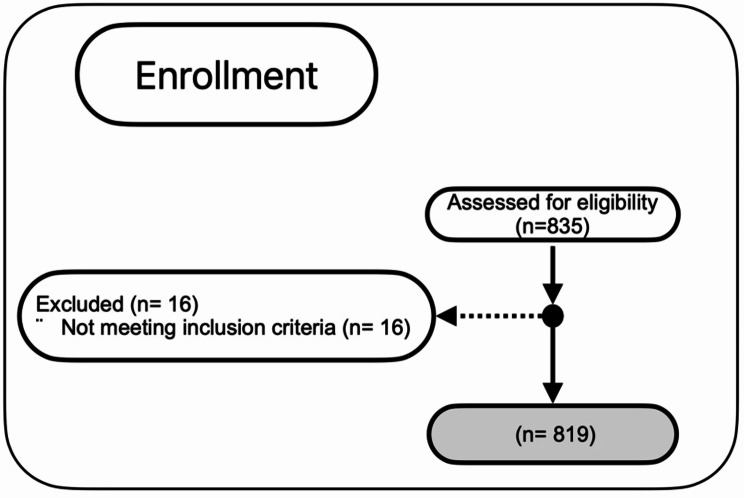
Enrollment process

The present study included a total of 819 community-dwelling older adults aged 60 years and above from the SNAC research center of Blekinge (SNAC-B) (Fig. 1). A total of 16 participants were excluded from the study due to living in a nursing home. The study adhered to the ethical principles outlined in the Declaration of Helsinki [11] and was approved by the ethics committee of Lund University (LU 128–00, LU 604–00). All participants received written information about the SNAC and provided written informed consent before entering the SNAC.

### Measures

#### Outcome

##### Five-time test of sit-to-stand time (STS)

The outcome was STS time as it has shown to be essential for older adults’ capability to live independently and is a validated measure for lower body strength [4]. Participants completed five timed attempts to rise from a designated chair as quickly as possible, with their completion time recorded in seconds [5]. All participants utilized the same procedure, and materials.

#### Physical activity and physical function

Following measures were self-rated: Physical activity, Activities of daily living, Having fallen in the preceding twelve months, and Outdoor walking. Whilst GRIPPIT and one-legged stand were objectively measured.

##### Physical activity

Frequency of performance of physical activities of perceived light to moderate intensity and vigorous intensity was categorized as less than once a week (never, once a month, two–three times a month) or at least once a week (at least once a week, every day).

##### Activities of daily living (KATZ-ADL index)

The KATZ-ADL index measures the ability of older adults and chronically ill patients to perform basic tasks without assistance [12]. The index assesses daily activities like hygiene, dressing, and toileting. It uses a 0–2 score (higher = more dependence) and in this study dichotomized to “dependent” (0) or “independent” [1]. This aligns with past Swedish studies on disability using KATZ-ADL index [13].

##### Having fallen in the preceding twelve months

Having fallen in the preceding twelve months was categorized as no (never) or yes (from once to more than four times).

##### Outdoor walking

Outdoor walking was categorized as dependent (using assistive device) or independent (not using any assistive device).

##### Grip strength

The GRIPPIT dynamometer was used to measure grip strength (force in newtons). Both hands was tested and the average results calculated [14].

##### One-leg stand

Participants balanced on one leg (barefoot, eyes open) for up to 60 s (three trials per leg, best time used). This common test measures static balance. This is a well-established and validated measure of static balance [15]. Normative values for people in three age groups (60–69, 70–79, 80+) was based on the findings of Springer, Marin [16].

#### Health-related measures

##### EuroQol (EQ-5D)

The EQ-5D, a self-reported measure [17], assesses health across five domains and generates a utility score (-0.59 to 1.0) reflecting health-related quality of life (HRQoL). Participants in this study also rated their health on a visual analogue scale (VAS) (0–100, best health = highest score) [17]. EQ-5D analysis used time trade-off values (from UK) with VAS data for Sweden, similar to past studies [18].

##### Depression

The Montgomery-Åsberg Depression Rating Scale (MADRS) is a subscale of the Comprehensive Psychopathological Rating Scale for the rating of depressive symptoms [19]. It includes 10 items and the total MADRS score ranges from 0 to 60, with higher values indicating depression. This approach to exploring depression among older adults has been used in previous research [20].

##### Sense of coherence

Sense of coherence (SOC) was assessed with the 13-item version of the SOC questionnaire [21]. The five negatively formulated items were reversed and scored from 13 to 91 (higher = better stress response), and Swedish norm values were used for dichotomization [22].

##### SF-12

The 12-item short form health survey (SF-12) was used to measure physical and mental health [23]. SF-12’s generic design allows it to track health in large populations. Individual item responses were weighted and then combined to create two summary scores (ranged 0-100): one for physical health and one for mental health [23].

#### Control factors

Age was categorized as 60–69 years, 70–79 years, or ≥ 80 years. Education level was categorized as “Up to complete primary school” (non-completed primary school up to completed primary school) and “Above primary school” (secondary school to graduate school). *Gender* was a dichotomous variable (man or woman), *Living alone* was categorized as no (all answers but living alone) or yes (living alone), *Economy* was dichotomous (not able to cover unexpected expenses of 14,000 Swedish krona (SEK) within a week or able to cover unexpected expenses of 14,000 SEK within a week).

Grip strength, HRQoL, MADRS, and EQ-5D were dichotomized as low (first quartile) or high (second–fourth quartile) in the descriptive part of the results and used in the continuous form in the multiple linear regression.

### Statistical analysis

Descriptive statistics for personal characteristics, and STS time were presented as frequencies and percentages for categorical variables. The chi-squared test were used to compare data on distribution of personal characteristics and STS time between groups [24]. In the crude analysis, we examined the bivariate association between STS time and the predictors using simple linear regression.

A multiple linear regression model including all variables utilizing backward elimination procedure was conducted. STS time acted as the outcome, and grip strength, age, health-related quality of life, having fallen within the preceding twelve months, physical activities of perceived light to moderate intensity, and education as significantly associated predictors. The model’s squared dependent variable resulted in normally distributed residuals, no heteroscedasticity, and low collinearity (all variance inflation factors were approximately 1). Block-wise linear regression was performed to assess the individual contribution of each predictor. The magnitude of the change in R-squared was used to assess the individual contribution of each predictor to the model, thereby providing insights into their relative importance [25]. The outcome of the regression model is presented with β, β’, confidence interval (CI), p value, and $\:{R}^{2},\:$as recommended for statistical interpretation [24, 25]. To emphasize both clinical and scientific impacts, unstandardized and standardized betas were analyzed. All tests were two-tailed with a significance level set at *p* = 0.05. Data were processed using IBM^®^ SPSS^®^ version 28.0.

## Result

The participants included 451 women (median age 72 years, range 36) and 368 men (median age 72 years, range 36). As shown in Table 1, the mean time of STS time was 11.9 s (standard deviation (SD) 4.2), with males being faster (11.2 s, SD 4.0) than females (12.5 s, SD 4.4). As regards age groups, 60–69-year-olds had the fastest STS time (10.0 s, SD 3.3), 70–79-year-olds were in the middle (12.2 s, SD 3.8), and participants aged 80 years and above had the slowest STS time (14.1 s, SD 4.8) (Table 1). A high value on grip strength, health-related quality of life (EQ-5D) physical health (SF-12), or mental health (SF-12), correlated with faster STS time. Whilst a low value on depression (MADRS) correlated with faster STS time. Among the participants, 76% were engaged in weekly physical activity of perceived light to moderate intensity, 54% engaged in weekly physical activity of perceived vigorous intensity. A share of 77% was independent in KATZ ADL index, 69% reached the age-related time limit for the one-leg stand, nearly 78% were able to walk outdoors independently and for 80% of the participants no falls had occurred in the last twelve months. (Table 1). More than 92% of the participants were able to cover unexpected expenses of 14,000 SEK within a week, 68% lived with someone, 65% had completed at least secondary school and 40% reached the age-related Swedish norm value indicating SOC.

**Table 1:** Sample characteristics and distribution of the results of STS time in seconds

	*N*	%	STS time (s, mean)	Standard deviation	*P* ^a^
Age (years) 70–79 ≥ 80 60–69	819 256 279 284	31.3 34.1 34.7	10.0 12.2 14.1	3.3 3.8 4.8	**< 0.001**
Gender Man Woman	819 368 451	44.9 55.1	11.2 12.5	4.0 4.4	**< 0.001**
Can you within a week cover unexpected expenses of 14,000 SEK? No Yes	653 51 602	7.8 92.2	12.5 11.8	4.7 4.2	0.883
Living alone No Yes	653 446 207	68.3 31.7	11.5 12.7	4.2 4.0	**< 0.001**
Education Primary school, left at age < 13 yrs. Secondary school, left at age 14–16 yrs. Upper secondary or vocational school, left at age 18–19 yrs. University	819 258 100 230 167	34.2 13.2 30.5 22.1	13.2 11.4 11.9 10.9	4.2 4.0 4.2 4.1	**< 0.001**
Outdoor walking Dependent Independent	818 177 641	21.6 78.4	15.6 11.5	4.8 4.0	**< 0.001**
Having fallen within preceding twelve months No. Yes.	800 642 158	80.2 19.8	11.9 12.0	4.2 4.2	0.329
Physical activities of perceived light to moderate intensity < Once a week ≥ Once a week	653 158 495	29.3 75.8	13.4 11.4	4.9 3.9	**< 0.001**
Physical activities of perceived vigorous intensity < Once a week ≥ Once a week	650 299 351	46.0 54.0	12.4 10.8	4.3 3.8	**< 0.001**
KATZ ADL index Dependent Independent	812 189 623	23.3 76.7	13.1 11.7	3.7 4.3	**< 0.001**
Grip strength ^b^ Low High	702 176 526	25.0 75.0	14.6 10.9	4.4 3.6	**< 0.001**
One-leg stand Not meeting age-related norm value Meeting age-related norm value	586 180 406	30.7 69.3	12.2 11.1	3.7 3.8	**0.007**
Sense of coherence < Swedish norm value ≥ Swedish norm value	638 381 257	59.7 40.3	12.0 11.8	4.2 4.1	**< 0.001**
Health-related quality of life (EQ-5D) ^b^ Low High	650 169 481	25.0 75.0	13.4 11.2	4.7 3.8	**< 0.001**
Physical health (SF-12) ^b^ Low High	634 158 476	25.0 75.0	11.7 12.0	4.0 4.3	0.766
Mental health (SF-12) ^b^ Low High	634 158 476	25.0 75.0	13.0 11.5	4.4 4.1	**0.012**
Depression (MADRS) ^b^ Low High	654 242 520	25.0 75.0	10.8 11.8	3.3 4.4	**< 0.001**
Tot	819^c^	100^c^	11.9	4.2	

^a^ p for the differences between groups as calculated with the chi-squared test

^b^ Linear variable with quartile range (≤ 25 and > 25)

^c^ Total numbers may not be equal to 819 and 100% due to missing data for some variables

### Crude analysis of predictors

In the crude analysis (Table 2), positive associations were found between STS time and age (β = 0.028), having fallen within the preceding twelve months (β = 0.027), gender (β = 0.199), living alone (β = 0.183), comprehensive psychopathological rating scale (β = 0.027), and physical health (β = 0.028). Negative associations was found between STS time and education (β = -0.216), physical activity of perceived light to moderate intensity (β =-0.276), physical activity of perceived vigorous intensity (β = -0.232), health-related quality of life (β = -0.591), one-leg stand (β = -0.008), grip strength (β = -0.002), economy (β = -0.078), outdoor walking (β = -0.560), capacity for activities of daily living (β = -0.223), sense of coherence (β = -0.021), and mental health (β = -0.021).

### Adjusted analysis of predictors

In the adjusted analysis (Table 2), the model explained 26.8% of the variance in STS time. Out of the seven predictors explained by β’, grip strength (β’ = -0.204; R^2^ = 12.0%), age (β’ = 0.202; R^2^ = 7.2%), and health-related quality of life (β’ = -0.192; R^2^ = 2.8%) explained STS time to the greatest degree.

Out of the seven predictors explained by β, health-related quality of life (β = -0.354, CI (-0.509)–(-0.199)), having fallen in the preceding twelve months (β = -0.222, CI (-0.365)–(-0.078)), and physical activities of perceived light to moderate intensity (β = -0.166, CI (-0.278)–(-0.053)) were most prominent in the model (Table 2).

**Table 2:** Crude and adjusted analysis

	Crude analysis				Adjusted analysis ^a^				
	β	β’	CI 95%	p	β	β’	CI 95%	p	R^2^
Variable									< 0.001
Age	0.028	0.417	0.985 − 1.679	**< 0.001**	0.014	0.202	0.007 − 0.021	**< 0.001**	0.072
Education	-0.216	-0.201	(-0.362) − (-0.161)	**< 0.001**	-0.114	-0.092	(-0.217) − (-0.011)	**0.031**	0.010
Physical activities of perceived light to moderate intensity.	-0.276	-0.195	(-0.390) − (-0.163)	**< 0.001**	-0.166	-0.121	(-0.278) − (-0.053)	**0.004**	0.013
Having fallen in preceding twelve months	0.027	0.016	(-0.100) − 0.154	0.680	-0.222	-0.127	(-0.365) − (-0.078)	**0.002**	0.011
Health-related quality of life (EQ-5D)	-0.591	-0.321	(-0.734) − (-0.448)	**< 0.001**	-0.354	-0.192	(-0.509) − (-0.199)	**< 0.001**	0.028
One-leg stand	-0.008	-0.337	(-0.009) − (-0.006)	**< 0.001**	-0.002	-0.099	(-0.010) − (-0.001)	**0.048**	0.007
Grip strength	-0.002	-0.375	(-0.003) − (-0.002)	**< 0.001**	-0.001	-0.204	(-0.002) − (-0.001)	**< 0.001**	0.120
Gender	0.199	0.170	0.111 − 0.288	**< 0.001**					
Economy	-0.078	-0.033	(-0.271) − 0.115	0.426					
Living alone	0.183	0.142	0.078 − 0.288	**< 0.001**					
Outdoor walking	-0.560	-0.278	(-0.707) − (-0.412)	**< 0.001**					
Physical activities of perceived vigorous intensity	-0.232	-0.187	(-0.332) − (-0.132)	**< 0.001**					
KATZ-ADL index	-0.223	-0.136	(-0.384) − (-0.098)	**< 0.001**					
Sense of coherence	-0.021	-0.018	(-0.120) − 0.077	0.669					
Physical health (SF-12)	0.028	0.238	0.018 − 0.037	**< 0.001**					
Mental health (SF-12)	-0.021	-0.190	(-0.030) − (-0.012)	**< 0.001**					
Depression (MADRS)	0.027	0.164	0.014 − 0.039	**< 0.001**					
Total variance explained (R^2^)									0.268

^**a**^ Only variables significantly associated with the outcome are presented. Variables are listed as selected during the backward elimination procedure in the multiple linear regression analysis

^b^ The square of the dependent variable “STS” is shown

Reference values: Education (0 = below secondary school, 1 = secondary school or more), Physical activities of perceived light to moderate intensity (0 = less than weekly engagement, 1 = weekly engagement), Having fallen in preceding twelve months (0 = no, 1 = yes), Gender (0 = man, 1 = woman), Economy (0 = not able to cover unexpected expenses of 14,000 SEK within a week, 1 = able to cover unexpected expenses of 14,000 SEK within a week), Living alone (0 = no, 1 = yes), outdoor walking (0 = dependent, 1 = independent), Physical activities of perceived vigorous intensity (0 = at least weekly engagement, 1 = weekly engagement), Capacity for activities of daily living (0 = dependent, 1 = independent)

## Discussion

With the aim to investigate the associations between STS time, self-rated physical activity, physical function, health-related quality of life, physical and mental health in community-dwelling older adults aged ≥ 60 years, we found predictors explaining 26.8% of the variance and the most impactful predictors. Our findings show that grip strength, age, and health-related quality of life were the most dominant predictors in explaining the variance in STS time. Furthermore, when considering the predictors that most strongly influenced the actual STS time, health-related quality of life, a history of falls in the preceding twelve months, and physical activity of perceived light to moderate intensity were the most distinguished.

In this study, the complex process of aging emerges as an interesting predictor of STS time. In short, as we get older, it takes a longer time to rise from sitting. This can be understood by the associations between aging and decline in physical function, with age-related sarcopenia being one contributing factor [8].

Grip strength has in previous research been shown to be a reliable indicator of overall physical function and health [8]. In our study, greater grip strength predicted faster STS time, which aligns with previous research regarding its relation to the physical function needed to independently stand up from a seated position [8]. Having the ability to stand on one leg is also a strong indicator of the physical function needed to independently stand up from a seated position [15]. Our study confirms that longer duration of one-leg balance predicts a faster STS time, as expected and supported by prior research on balance and physical function in older adults [15].

Health-related quality of life was used to identify how older adults’ self-rated quality of life was weighted against a health-economy perspective. Overall high physical and mental health and quality of life have in previous research been found to be related to being more physically active and having greater physical function [5]. Our findings align with these studies, as a higher health-related quality of life predicted a shorter STS time.

People who reported more light to moderate physical activity had faster STS times in our study. This matches other research showing physical activity improves strength and rising ability [26].

A history of falling have been associated with slower STS time [7]. Our study found a surprising result: prior falls associated to faster STS time. This might be because we only considered those who had not fallen vs. those who had. A single fall might not trigger fear in older adults, which is important to bear in mind since fear of falling have shown association with lower levels of daily physical activity among older adults [27, 28]. In other words, physical activity could cause, and prevent falls. This paradox of physical activity as both a protective factor and a risk factor for falls is nothing new [29]. However, our finding adds important notes regarding the assumption that older adults who have fallen also tend to be more physically functional.

### Strengths and limitations

#### Design

This investigation centers on the analysis of data procured from the SNAC. Leveraging existing data is a significant advantage in terms of efficiency. The SNAC used rigorous methodologies, resulting in high-quality and dependable data [10]. This reduces concerns about potential biases or inconsistencies that might have arisen during primary data collection [30]. Pre-existing data may not perfectly align with every research goals and might have biases. Careful data extraction, expert consultation, and critical evaluation of biases helped ensure robust analysis. Self-reported data, such as HRQoL, may be underreported, but we addressed this limitation by repeatedly discussing possible outcomes and variations. A critical evaluation of these potential biases were conducted and was crucial to ensure the robustness of our analysis [30]. Lastly, the data might not reflect the most recent trends or developments, as they were taken from a longitudinal aging study. Therefore, the variables underwent a thorough review by the research team before being analyzed, with great emphasis put on the context of the data collection period in relation to the research question.

This study benefits from a robust population-based design, employing a large and nationally representative sample of older adults. This approach enhances the generalizability of the findings to the broader population [30].

#### Variables

The primary outcome was STS time, a validated objective measure for identifying obstacles in physical function based on the time it takes to stand up from sitting [4]. However, it is important to highlight that physical function is more than just this one measure, and that the gold standard for assessing physical function and physical activity in older adults is under constant development [31].

As shown in Table 1 the variables Can you within a week cover unexpected expenses of 14,000 SEK? Living alone, Physical activities of perceived light to moderate intensity, Physical activities of perceived vigorous intensity, Sense of coherence, Health-related quality of life, Physical health, Mental health, Comprehensive psychiatric scale, One-leg stand, and Grip strength all had missing values ranging between 14% and 22%. Contact was made with the database manager of SNAC-B to ensure that no erroneously processed data were used in this study.

In relation to previous falls, it is normal to include fear of falling as a variable to assess physical function, but while fear of falling is a potentially significant variable, it is not currently included in the SNAC data. Furthermore, the analysis could have benefited of including information regarding the location of the fall, such as it occurred inside or outside of the house. While gait (walking speed) was considered due to its strong link to morbidity and sedentary time, it was not available as a variable in the 2013 database, it was first implemented in 2015. Thus, to include more participants, gait was excluded. To improve the analysis of the multiple regression and provide the best fit, STS time counts were squared. Furthermore, 174 older adults were excluded from the multiple regression model to increase fit [24].

## Conclusion

The model of predictors provides a new understanding of STS time among community-dwelling older adults. The association between faster STS time and grip strength offers valuable insights for clinical practitioners regarding the connectivity of lower and upper body strength. Health-related quality of life was a key factor in predicting STS time, with even small changes impacting performance. Therefore, regular assessment and monitoring of grip strength and health-related quality of life in older adults can be beneficial findings for clinical practitioners developing interventions that aim to maintain physical function among older adults. Future research should investigate the detailed association between fall history and faster STS time, and explore how grip strength and health-related quality of life impact sedentary behavior among older adults.

## Data Availability

The datasets generated and/or analyzed during the current study are not publicly available due to European governing laws and agreements between participants and the research group regarding data management. These datasets are archived with the Department of Health at Blekinge Institute of Technology (BTH), Sweden. However, they are available from the authors upon reasonable request and with permission from the Department of Health at BTH.

## Abbreviations

β: Unstandardized regression coefficient
β’: Standardized regression coefficient
CI: Confidence interval
EQ-5D: EuroQol
HRQoL: Health-related quality of life
MADRS: Montgomery-Åsberg Depression Rating Scale
p: p value
R^2^: Coefficient of determination for variance in linear regression model
SD: Standard deviation
SEK: Swedish Krona
SNAC: Swedish National Study on Aging and Care
SOC: Sense of coherence
STS: Sit-to-stand
VAS: Visual analogue scale
